# Foreign Body Presenting as Golden Hypopyon

**DOI:** 10.3390/surgeries6030068

**Published:** 2025-08-05

**Authors:** Anas Alkhabaz, Lucie Y. Guo, Charles DeBoer

**Affiliations:** Byers Eye Institute, Department of Ophthalmology, Stanford University School of Medicine, Palo Alto, CA 94303, USA

**Keywords:** intraocular foreign body, hypopyon, anterior chamber

## Abstract

**Background/Objectives:**

Penetrating intraocular foreign bodies (IOFBs) are ocular emergencies, often leading to preventable vision loss. This case report highlights a unique presentation of a work-related penetrating IOFB that mimicked a golden hypopyon.

**Methods:**

A 35-year-old male presented to the emergency department with sudden-onset pain and vision loss in the left eye while he was cutting a tree with metallic scissors. He had a visual acuity of 20/30 in the right eye and counting fingers in the left eye. A dilated slit-lamp examination and CT scan confirmed the presence of a 6–8 mm metallic IOFB in the anterior chamber, with no involvement of the lens or the posterior segment. Surgical removal was performed.

**Results:**

The metallic IOFB was removed surgically with IOFB forceps using a single paracentesis. The patient reported resolving pain and regained baseline visual acuity of 20/20 postoperatively, which remained stable at one-month follow-up.

**Conclusions:**

This case illustrates the successful surgical management of a penetrating metallic IOFB with a unique presentation mimicking a hypopyon. Emphasis on unique presentations of IOFBs can aid in timely management, ultimately reducing the risk of complications.

## 1. Introduction

Penetrating intraocular foreign body (IOFB) is an ocular emergency and a common cause of preventable visual impairment. It can disrupt delicate structures in the eye and cause open globe injuries. The presence of IOFB can be complicated by a range of blinding sequalae such as retinal detachment, choroidal hemorrhage, and endophthalmitis [1,2]. IOFBs comprise 16% to 41% of open globe etiologies and most commonly occur in males, particularly from work-related injuries [3–5]. The visual outcome in these patients often depends on several factors including the initial visual acuity, IOFB size and location, entry wound size and location, presence of relative afferent pupillary defect (RAPD), and concomitant complications (i.e., endophthalmitis) [5]. Surgical removal is often necessary for penetrating IOFBs. The surgical approach depends on the location, material, and size of the IOFB. Overall, metallic IOFB are the most common form and are more likely to involve the posterior segment of the eye, which requires a more complex surgical management. On the contrary, IOFBs in the anterior chamber can be managed with less invasive techniques [1,5]. Herein, we present a surgical video of IOFB removal in a unique case of work-related penetrating IOFB mimicking a golden hypopyon.

## 2. Case Presentation

We report a case of a 35-year-old male with no past medical history who presented to the emergency department with sudden-onset pain and vision loss in the left eye after cutting a tree with metallic scissors. Upon presentation, the patient had a visual acuity of 20/30 and counting fingers (CF) at 1 foot in the right and left eye, respectively.

**Figure 1.**
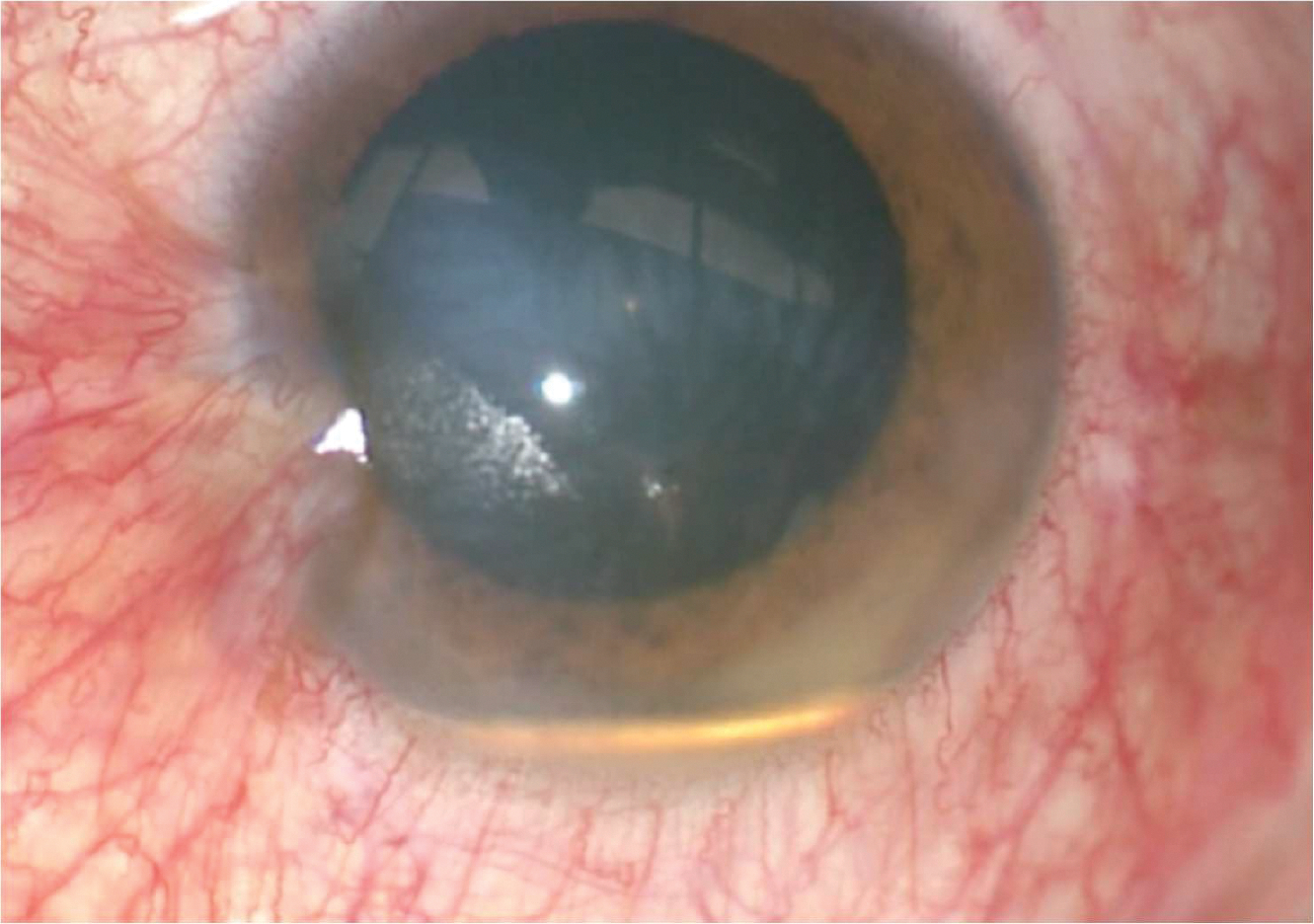
Photograph of the anterior segment of the left eye.

**Figure 2.**
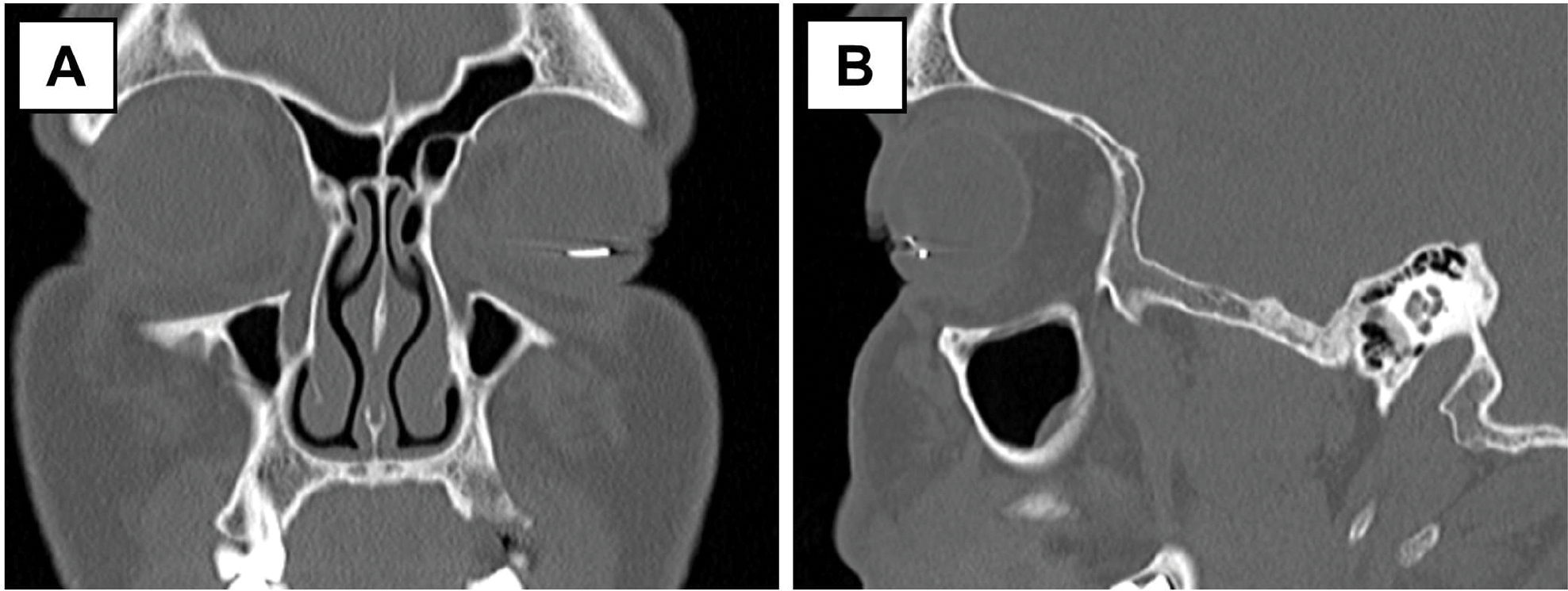
CT scan of the orbit in the coronal (**A**) and sagittal (**B**) views demonstrates a hyperintense signal corresponding to the metallic foreign body in the anterior segment of the left eye.

On the initial brief examination of the eye, the ocular appearance mimicked a golden-colored hypopyon. However, on slit-lamp examination, a metallic piece was appreciated with a small space between the piece and the bottom of the anterior chamber (Figure 1). Conjunctival hyperemia and a 2 mm self-sealed corneal laceration (with a negative Seidel sign) were also noted. He had no RAPD. CT orbits revealed a metallic intraocular foreign body resting in the anterior chamber of the left eye (Figure 2).

A metallic 6–8 mm linear intraocular foreign body with adjacent inflammatory fibrinous material was removed operatively from the anterior chamber through a single paracentesis using IOFB forceps. The video of the IOFB removal procedure is available online (Video S1).

**Figure 3.**
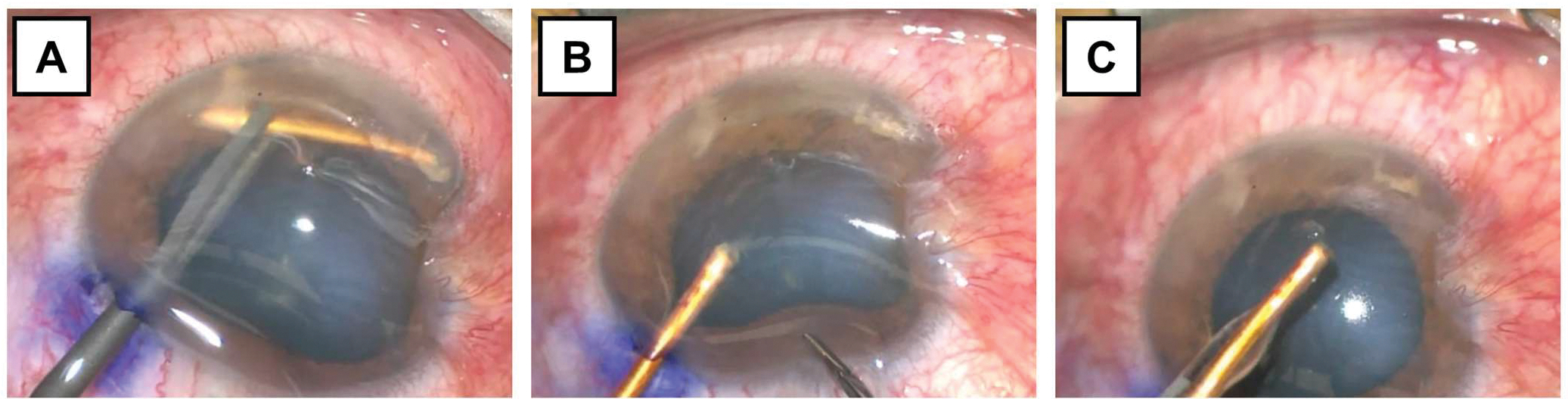
Sequential intraoperative photographs of the left eye highlighting the extraction of the intraocular foreign body from the anterior chamber using a single paracentesis at 1:00 o’clock. (**A**) Using the IOFB forceps to grasp the foreign body in the anterior chamber (**B**) Removal of the foreign body through the paracentesis and (**C**) a photograph of the extracted foreign body in front of the eye.

Systemic antibiotics were given prior to the surgery. In addition, vancomycin (1 mg in 0.1 mL), voriconazole (100 μg in 0.1 mL), and ceftazidime (2.25 mg in 0.1 mL) were injected intravitreally. A paracentesis wound was made at 1:00 (Figure 3A). Viscoelastic was injected into the anterior chamber to protect the corneal endothelium and deepen the chamber to allow manipulation of the IOFB. IOFB forceps were used to remove the metallic object (Figure 3B,C). A 10–0 nylon suture was placed over the self-sealed corneal wound and over the paracentesis wound at 1:00. Remarkably, the lens was intact, and there was no posterior segment involvement.

On postoperative day 1, the patient reported resolving pain. His vision remained CF at 2 feet due to the central corneal haze at the entry wound. On postoperative day 7, the patient regained his baseline visual acuity of 20/20 in the left eye, which remained stable one month later. The corneal suture was removed at 1 month to reduce the risk of astigmatism or corneal scarring.

## 3. Discussion

IOFBs can lead to prominent visual impairment if not managed promptly. Urgent surgical removal can be necessary to preserve vision. This case illustrates the surgical intervention in a unique presentation of a hypopyon-mimicking IOFB resulting from work-related ocular injury. Prognosis in these patients can be variable and depends on patient factors, wound factors, and associated complications. Improved surgical techniques can particularly aid in achieving better outcomes. In this case, the patient presented with a severe decline in visual acuity, a 6–8 mm IOFB with a 2 mm entry wound, and no RAPD, with the IOFB lodged in the anterior chamber. In addition, there were no related complications in the posterior segment. This helped restore baseline visual acuity in this patient with a minimally invasive surgical approach, as shown in the Supplementary Video.

Confirming the presence and the location of an IOFB is essential for management. Initial evaluation is often performed with slit-lamp examination and dilated fundus examination. Examination may be limited in the setting of globe violation. CT scan of the orbits is non-contact, can be used to detect radiopaque IOFBs, and provides information regarding the size, shape, composition, and localization of the IOFB [5–7]. This is considered the standard modality to evaluate for metallic IOFB due to its versatility. Plain-film X-ray is not used often due to reduced diagnostic ability relative to CT [6]. MRI may be used to evaluate when IOFB is suspected but was not found on CT. Of note, MRI is contraindicated in the setting of metallic IOFB as it can cause dislodgement. Ultrasonography can be used for the diagnosis of IOFB. B-scan ultrasound can be particularly helpful to determine the extent of damage and posterior segment complications (i.e., choroidal, vitreous hemorrhage, or retinal detachment). However, it must be used judiciously in experienced hands, as in open globe injury, it can induce pressure and expulsion of ocular content [5,7]. UBM has an excellent ability to detect IOFBs in the anterior segment, but due to the pressure applied during the technique, is most suited after the globe injury has been stabilized [8,9].

The goal of surgery is to remove the IOFB and restore ocular anatomy, with minimal complications. Different approaches are taken based on the location, material, and size of the IOFB. In terms of location, IOFBs in the posterior segment are usually removed with pars plana vitrectomy. Associated retinal detachment is treated with a combination of scleral buckle and/or pars plana vitrectomy with gas or oil tamponade. In the anterior segment, the IOFB can be removed with a limbal incision and using viscoelastic solution to maintain the anterior chamber depth and protect the corneal endothelium. For small metallic objects, an intraocular magnet can be used. Nonmagnetic or larger objects are removed with intraocular forceps. If the lens is involved, lensectomy is performed via phacoemulsification, pars plana lensectomy, or a manual extracapsular cataract extraction (ECCE) [5,7]. In our patient, we used a paracentesis 180 degrees from the IOFB and removed the IOFB directly with forceps. The IOFB was grasped from the end to assist with alignment of the forceps, facilitating removal. If alignment was not possible, the wound would have had to be expanded, or a second main corneal incision would have been necessary. Yet, working through the paracentesis helped improve anterior chamber stability and assisted with avoiding contact with the lens compared with a larger incision. Another possible approach would be to float the IOFB out of the paracentesis using a no-touch technique [10]. This approach is often used with dexamethasone implant as they fragment when grasped with forceps. In our situation, the IOFB was a small cylinder and mechanically strong enough to be grasped directly with forceps.

## Supplementary Material

Supplementary Material - Surgical VIdeo

**Supplementary Materials:** The following supporting information can be downloaded at: https://www.mdpi.com/article/10.3390/surgeries6030068/s1. Video S1: Surgical removal of the intraocular foreign body.

## Data Availability

The data presented in this study are included in the article/Supplementary Material. Further inquiries can be directed to the corresponding author.

## Abbreviations

The following abbreviations are used in this manuscript:

IOFB: Intraocular foreign body
RAPD: Relative afferent pupillary defect
CF: Counting fingers
